# The Infection of the Japanese Encephalitis Virus SA14-14-2 Strain Induces Lethal Peripheral Inflammatory Responses in IFNAR Deficiency Mice

**DOI:** 10.3389/fmicb.2021.823825

**Published:** 2022-03-03

**Authors:** Juan Liu, Wenxian Jing, Yongxiang Fang, Xiaobing He, Guohua Chen, Huaijie Jia, Jingyu Wang, Zhizhong Jing

**Affiliations:** ^1^State Key Laboratory of Veterinary Etiological Biology, Key Laboratory of Veterinary Public Health of Agriculture Ministry Lanzhou Veterinary Research Institute, Chinese Academy of Agricultural Sciences, Lanzhou, China; ^2^College of Veterinary Medicine, Northwest A&F University, Yangling, China

**Keywords:** Japanese encephalitis virus, SA14-14-2 strain, IFNAR deficiency mice, infection, immunopathogenesis, peripheral inflammation

## Abstract

The Japanese encephalitis virus (JEV) is a leading cause of mosquito-borne viral encephalitis worldwide. Clinical symptoms other than encephalitis, on the other hand, are substantially more prevalent with JEV infection, demonstrating the relevance of peripheral pathophysiology. We studied the peripheral immunopathogenesis of JEV using IFNAR deficient (IFNAR^–/–^) mice infected with the SA14-14-2 strain under the BSL-2. The body weight and survival rate of infected-IFNAR^–/–^mice decreased significantly. Infected-IFNAR^–/–^mice’s liver and spleen demonstrated obvious tissue damage and inflammatory cell infiltration. There was also extensive viral replication in the organs. IFN-α/β protein expression was dramatically elevated in peripheral tissues and serum, although the related interferon-stimulated genes (ISGs) remained low in the spleen and liver of infected-IFNAR^–/–^animals. Consistently, the differentially expressed genes (DEGs) analysis using RNA-sequencing of spleens showed inflammatory cytokines upregulation, such as IL-6, TNF-α, and MCP-1, and IFN-γ associated cytokine storm. The infiltration of macrophages and neutrophils in the spleen and liver of SA14-14-2-infected IFNAR^–/–^ mice was dramatically elevated. However, there was no significant difference in tissue damage, viral multiplication, or the production of IFNα/β and inflammatory cytokines in the brain. Infection with the JEV SA14-14-2 strain resulted in a lethal peripheral inflammatory response and organ damage without encephalitis in IFNAR^–/–^ mice. Our findings may help shed light on the peripheral immunopathogenesis associated with clinical JEV infection and aid in developing treatment options.

## Introduction

Japanese encephalitis virus (JEV), as positive single-stranded RNA virus, along with dengue virus (DENV), West Nile virus (WNV), Yellow fever virus (YFV), and Zika virus, mainly makes up of genus *Flavivirus* (Chambers et al., 1990; Unni et al., 2011). JEV affects the central nervous system (CNS) and causes 50,000–70,000 viral encephalitis cases with a high case fatality rate of 30–50% worldwide (Ghosh and Basu, 2009; Misra and Kalita, 2010). JEV is one of the mosquito-borne flaviviruses and is transmitted by culex. After the host is bitten by a mosquito infected with JEV, the virus begins to replicate, migrates through the periphery, such as draining lymph nodes, spleen, and kidney, and even crossing the blood-brain barrier (BBB) into the CNS (King et al., 2007; Dutta and Basu, 2014). Bovine and water birds amplify host supply virus in the blood for feeding mosquitoes, and finally, JEV infects dead-end host humans (Turtle and Solomon, 2018). Japanese encephalitis (JE) is endemic to the entire Asia-Pacific region and has recently spread to new geographical locations due to the expansion of the mosquito territory (Erlanger et al., 2009; Connor and Bunn, 2017).

The clinical symptoms initiate fever, headache, vomiting, and progress to encephalitis symptoms, including movement disorders, neurologic deficits, and seizures (Solomon et al., 2002; Misra and Kalita, 2010). These symptoms result from severe immune injury in CNS. Pro-inflammatory cytokines like TNF-α, IL-6, IFN-α, and IL-12, chemokines like CCL2 and CCL5, and RANTES were secreted in large quantities during JEV infection associated with increased mortality mice (Ghoshal et al., 2007; Chen et al., 2010). Although severe encephalitis is the significant symptom of JE, most JEV infections either develop asymptomatic or subclinical infections (Connor and Bunn, 2017; Sharma et al., 2021). Less than 1% of individuals infected will progress to encephalitis, and about 10% of the infected develop minor illnesses (Halstead and Jacobson, 2013). JEV replication in monocytes and polymorphonuclear cells of peripheral tissues can provoke peripheral antiviral immune responses, eliminating most viruses (King et al., 2007), which suggests that the JEV replication and transmission are restricted by the peripheral inflammatory response (Sharma et al., 2021). However, very little is known about the peripheral immunopathogenesis of JEV infection. Existing knowledge comes from limited natural human infection and animal models of neurological disease. In patients with JEV infection, renal dysfunction, derangements in liver function, and thrombocytopenia are observed apart from the CNS damage (Kumar et al., 2006; Patgiri et al., 2014). The sharp increase of pro-inflammatory cytokines, especially in the spleen and serum, was detectable in the neurological disease mice models (Yang et al., 2011; Calvert et al., 2014).

Recently, the peripheral immunopathogenic of some flavivirus has been reported in a viscerotropic disease model. Because of flavivirus’s ability to promote complete and effective type I interferon responses, the immunocompromised mice, especially IFN deficient mice, were more widely used as animal models (Samuel and Diamond, 2005; Clyde et al., 2006; Lazear et al., 2016). Using AG129 mice (deficient in both IFN-α/β and IFN-γ receptor) as the model, virulent dengue virus (DENV) infection induces lethal, acute, disseminated cytokine storm without the neurologic disease (Sarathy et al., 2015). In the A129 (deficient in the IFN-α/β receptor) mouse model for studying viscerotropic disease, the virulent yellow fever virus (YFV) attack causes a lethal peripheral inflammatory response and severe pathology in visceral organs, especially the liver and spleen (Meier et al., 2009). These animal models of viscerotropic disease developed more closely, resembling most human infections.

Vaccination is an effective way to prevent encephalitis. The live attenuated vaccine (SA14-14-2 strain) has been developed successfully in China and is widely used to defend against JEV with an excellent safety profile and remarkable efficacy during large-scale vaccination programs (Yu, 2010; Hegde and Gore, 2017). The SA14-14-2 virus was generated from the wild strain SA14 and presented stable attenuation and high immunogenicity characteristics (Yu, 2010). Although current vaccines are available, limited vaccination coverage and very few clinical therapies have made the burden of JE disease high. It is essential to clarify peripheral pathogenesis mechanisms governing the clinical presentations during JEV infection without encephalitis. However, the mechanism research of virulent JEV is restricted to BSL-3 containment facilities for prevention and treatment. Using the stable attenuation and safety of the SA14-14-2 strain, developed in a flavivirus encephalitic animal model, makes the JEV infection research feasible in common biocontainment facilities (Calvert et al., 2014).

The study focused on peripheral inflammations without viral encephalitis in immunocompromised mice infected with the JEV SA14-14-2 strain, which lacked critical molecule involved in type I IFN (IFN-I) signaling transduction. IFNAR^–/–^ mice appeared with a lethal peripheral inflammatory response and exhibited viscerotropic JEV infection causing severe histopathology in the liver and spleen. Our results suggested that IFNAR^–/–^ mice infected with SA14-14-2 strain may be developed as a model to investigate peripheral immunopathogenesis of JEV under BSL-2 containment, which would contribute insight into the pathogenic mechanism and therapeutic studies of JEV infection.

## Materials and Methods

### Virus, Reagents, and Cells

JEV SA14-14-2 strain was propagated in BHK-21 cells, which were grown and maintained in Dulbecco’s Modified Eagle’s Medium (DMEM, Hyclone, Logan, UT, United States) supplemented with 2% heated-inactivated fetal bovine serum (Gibco, Invitrogen, Carlsbad, United States) at 37°C with 5% CO_2_. Virus titers were determined by a cytopathic assay using BHK21 cells, and aliquots of virus stocks were stored at −80°C.

### Mouse Experiments

Female C57BL/6 mice (B6 strain) aged between 6 and 8 weeks old were obtained from Laboratory Animal Center of Lanzhou Veterinary Research Institute (LVRI), Chinese Academy of Agriculture Science (CAAS). *Ifnar*^–/–^ mice were purchased from Jackson Laboratory. Subsequently, these mice were bred at the Laboratory Animal Centre of LVRI, CAAS.

Three groups of mice were inoculated intraperitoneally (i.p.) with 10-fold dilutions of virus (5 × 10^6^–5 × 10^4^ plaque-forming units [PFU]/mice) for survival rate determination. Mice aged between 6 and 8 weeks were challenged with 5 × 10^6^ PFU of virus per mouse, i.p., to record symptoms and body weight gain each day for 7 days following infection. Meanwhile, tissues, and serum were collected at 4 days post-infection (dpi) for use in Polymerase Chain Reaction (PCR) detection, plaque assay tested for viral burden, Enzyme-Linked Immunosorbent Assay (ELISA), and flow cytometry analysis.

### RNA Sequencing and Bioinformatics

The total RNA isolated from the spleen (*n* = 3 per group) was extracted using the trizol reagent (Invitrogen). The transcriptome sequencing and analysis were conducted by OE Biotech Co., Ltd. (Shanghai, China). According to the manufacturer’s protocol, the libraries were constructed using TruSeq Stranded mRNA LT Sample Prep Kit (Illumina, San Diego, CA, United States) and sequenced on an Illumina HiSeq X Ten platform. The DESeq (2012) R package (Yi et al., 2019) analyzed the differential genes expression. The significantly differential genes expressions were selected with the threshold of *P*-value<0.05 and log2|foldchange| ≥ 1. The expression pattern of genes in different groups and samples was demonstrated using hierarchical cluster analysis of differentially expressed genes (DEGs). GO enrichment analysis of DEGs was performed using R based on the hypergeometric distribution.

### RNA Preparation and Real-Time Quantitative PCR

Total RNA was extracted from tissue samples using a TransZol Up Plus RNA Kit (TransGen Biotech; Beijing, China). First-strand cDNA was synthesized using TransScript One-Step gDNA Removal and cDNA Synthesis SuperMix (TransGen Biotech) with 1 μg of total RNA. Then, the resulting cDNA was diluted 10-fold for subsequent real-time fluorescence quantitative PCR (qPCR). A Quantinova SYBR Green PCR Kit (Qiagen; Hilden, Germany) was used for qPCR with a Two-Step Real-Time PCR Detection System (Bio-Rad, United States) following the manufacturer’s instructions. The primer sequences used for the qPCR are shown in Supplementary Table 1. Data were normalized to the mRNA expression levels of the housekeeping gene β-actin. The 2^–ΔΔCt^ method was used to determine the relative gene expression.

### Histological Analysis

Samples from different organs were fixed in 10% neutral buffered formalin solution and then were embedded in paraffin. Sections (4-μm thick) of paraffin-embedded specimens were cut and stained with hematoxylin and eosin (H&E) for histopathological analyses.

### Cytokine Concentration Measurement

The tissues were lysed (1:10, w/v) in RIPA (Solarbio Life Sciences; Beijing, China) containing protease inhibitors to quantify the protein production of IFN-α, IFN-β, IL-6, and TNF-α from mouse organs. The supernatants from these tissue lysates were examined by ELISA, using a VeriKine Mouse IFN Alpha ELISA Kit (PBL Interferon Source), Legend Max Mouse IFN-β ELISA kit (BioLegend; CA, United States), Legend Max Mouse IL-6 ELISA kit (BioLegend), or Legend Max Mouse TNF-α ELISA kit (BioLegend). To further quantify the protein production of the major inflammatory cytokines, the supernatants from tissues lysates (brain, spleen, and liver) and serum were examined by flow cytometry, using a LEGENDplex™ Mouse Inflammation Panel (13-plex) with a filter plate (BioLegend), following the manufacturer’s instructions.

### Flow Cytometry

The cellular fractions of liver homogenates were re-suspended in 40% percoll (GE Life Sciences; Marlborough, MA, United States) in PBS and under-laid with 70% percoll. After centrifugation, liver leucocytes were isolated and collected from the 40–70% interface for FACS staining.

Single-cell suspensions prepared in FACS buffer (1% BSA in PBS) were blocked with anti-mouse CD16/32 antibody (Biolegend) to prevent non-specific binding. These samples were then incubated with antibodies including APC-Cy7 anti-CD45, PerCP-Cy5.5 anti-CD4, FITC anti-CD8, PerCP-Cy5.5 anti-CD11b, APC anti-F4/80, AF488 anti-Ly6G, and PE anti-NK1.1 (all purchased from Biolegend). The cells used for flow cytometric analyses were acquired using a BD Fortessa flow cytometer (BD Biosciences; San Jose, CA, United States). The results were analyzed using FlowJo software (TreeStar; Ashland, OR, United States).

### Statistical Analysis

Data were expressed as the means ± SD. We used the log-rank test for survival experiments, and other data were analyzed using a Student’s *t*-test (GraphPad Prism 5). In all figures, nd stands for not detected and ns stands for not significant; *P ≤ 0.05; ^**^P ≤ 0.01; and ^***^P ≤ 0.001.

## Results

### High Titer Japanese Encephalitis Virus SA14-14-2 Strain Infection Is Fatal to IFNAR^–/–^ Mice

**FIGURE 1:**
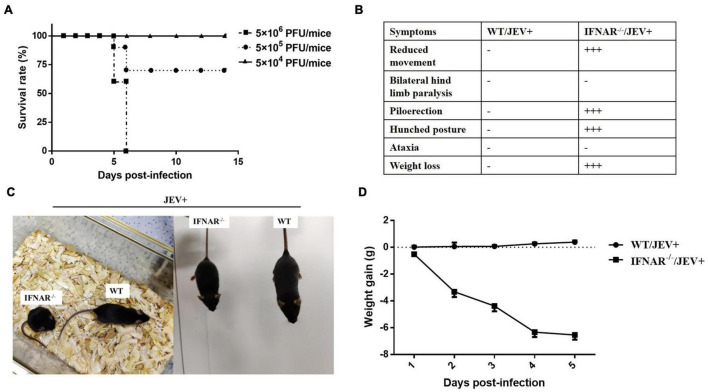
Mice lacking IFNAR were susceptible to JEV vaccine strain SA14-14-2. **(A)** Survival curves for IFNAR^–/–^ mice (*n* = 10) intraperitoneally infected with 10-fold dilutions of JEV vaccine strain SA14-14-2 (5 × 10^6^ PFU/mice, 5 × 10^5^ PFU/mice, and 5 × 10^4^ PFU/mice). Mice were monitored daily for up to 14 days. **(B)** The clinical presentation and **(C)** representative images of wild-type (WT) and IFNAR^–/–^ mice intraperitoneally infected with SA14-14-2 (5 × 10^6^ PFU/mice) at 4 days post-infection (dpi). −: no significant clinical change. +++: very significant clinical change. **(D)** Weight gain of JEV SA14-14-2 (5 × 10^6^ PFU/mice) infected wild-type (WT) and IFNAR^–/–^ mice (*n* = 6 per group).

To explore whether mice defective in interferon responses are susceptible to infection with SA14-14-2 strain, IFNAR^–/–^ mice were challenged intraperitoneally inoculated with 10-fold serial dilutions of virus ranging from 5 × 10^6^ to 5 × 10^4^ PFU/mice. The morbidity occurred in a dose-dependent manner, and IFNAR^–/–^ mice received 5 × 10^6^ PFU/mice of virus developed symptoms first with mortality up to about 40% at 5 dpi, 100% at 6 dpi (Figure 1A). The clinical presentation of infected IFNAR^–/–^ mice exhibited greatly reduced activity, piloerection, and hunched posture by 3–4 dpi infected with 5 × 10^6^ PFU/mice (Figure 1B). Interestingly, the IFNAR^–/–^ mice did not exhibit typical neurologic signs at the agonal stage, such as ataxia and hind-limb paralysis (Figure 1C). Furthermore, IFNAR^–/–^ mice presented a continuous body weight loss (Figure 1D). In contrast, WT mice remained in good condition and maintained their bodyweight gain following infection. Similar and relatively weaker clinical symptoms were observed with a dose of 5 × 10^5^ PFU/mice, and no significant symptoms were observed with a dose of 5 × 10^4^ PFU/mice (data not shown). These results indicated that the susceptibility and lethality of IFNAR^–/–^ mice increased dose-dependent to SA14-14-2, and IFNAR was indispensable for the attenuation of the SA14-14-2 strain *in vivo*.

### Virus Distribution and Pathological Changes in Organs of SA14-14-2 Strain-infected IFNAR^–/–^ Mice

**FIGURE 2:**
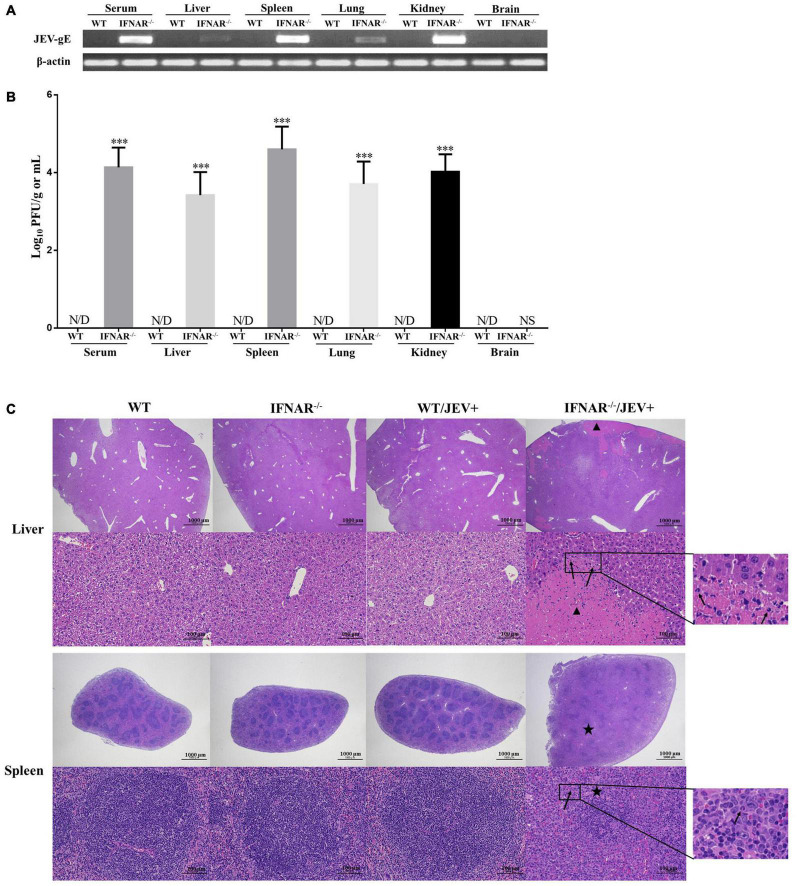
Virus distribution and pathological changes of liver and spleen from SA14-14-2 strain infected- and uninfected-WT and IFNAR^–/–^ mice. H&E; 20 × and 200 ×. **(A)** WT and IFNAR^–/–^ mice were intraperitoneally infected with JEV SA14-14-2 strain at 4 days post-infection (dpi), followed by PCR for the gE RNA levels in indicated organs and serum (*n* = 4 per group). **(B)** Corresponding JEV SA14-14-2 strain load in indicated organs and serum was analyzed by using plaque-forming unit counts. Error bars represent the standard deviation of triplicate measurement (*n* = 4 per group). **(C)** Representative pictures of tissues from the liver and spleen collected at 4 days post-infection (dpi) and stained with hematoxylin and eosin. Extensive necrosis (triangle) and infiltration of neutrophils (arrows) were detected in the liver, and severe lymphocytopenia (asterisk) and infiltration of neutrophils (arrows) were detected in the spleen. ^***^P < 0.001. NS, no significance. N/D, not detectable.

Next, to observe the pathogenicity characterization of the SA14-14-2 strain in IFNAR^–/–^ mice, the viral distribution and pathological changes were determined using a high titer (5 × 10^6^ PFU/mice) SA14-14-2 infection. The tissue distribution of the SA14-14-2 virus in WT and IFNAR^–/–^ mice was determined by real-time PCR and plaque assay. However, the Cq values of most samples exceeded 35 cycles (data not shown). Then, the PCR products were analyzed by using agarose gel electrophoresis. Both JEV-E RNA (Figure 2A) and infectious virus (Figure 2B) were detectable in the serum, liver, spleen, lungs, and kidney, but not the brain of IFNAR^–/–^ mice infected with SA14-14-2 by PCR and plaque assay at 4 dpi. However, neither JEV RNA nor live JEV was detected in the tested organs of WT mice infected with the SA14-14-2 strain. While SA14-14-2 virus amplified extensively in the visceral tissues and serum, replication of SA14-14-2 was still profoundly restricted in IFNAR^–/–^ mice. Titration of the spleen reached the highest level only with average titers of 4.6 log_10_PFU/ml (Figure 2B). These results indicated SA14-14-2 strain infection was fatal; meanwhile, it maintained stable neuroattenuation for IFNAR^–/–^ mice.

Histological analyses were performed on the organs from SA14-14-2-infected IFNAR^–/–^ mice at 4 dpi, where severe pathological changes were caused in the spleen and liver. Substantial amounts of focal necrosis and neutrophils were observed in the liver. Moreover, the spleen of these mice showed obvious enlargement and lymphoid depletion with infiltrating neutrophils (Figure 2C). However, no significant pathological changes were observed in the other tested organs, including neuron damage and gliocyte hyperplasia in the brain (Supplementary Figure 1). Tissues harvested from SA14-14-2 virus-infected WT mice or uninfected WT and IFNAR^–/–^ mice appeared normal (Figure 2C and Supplementary Figure 1). These results suggested that SA14-14-2 strain infection induced severe peripheral pathological changes, especially in the spleen and liver, in IFNAR^–/–^mice instead of the typical virus encephalitis.

### Transcriptional Profiles of the Spleen in SA14-14-2-Infection IFNAR^–/–^ Mice

**FIGURE 3:**
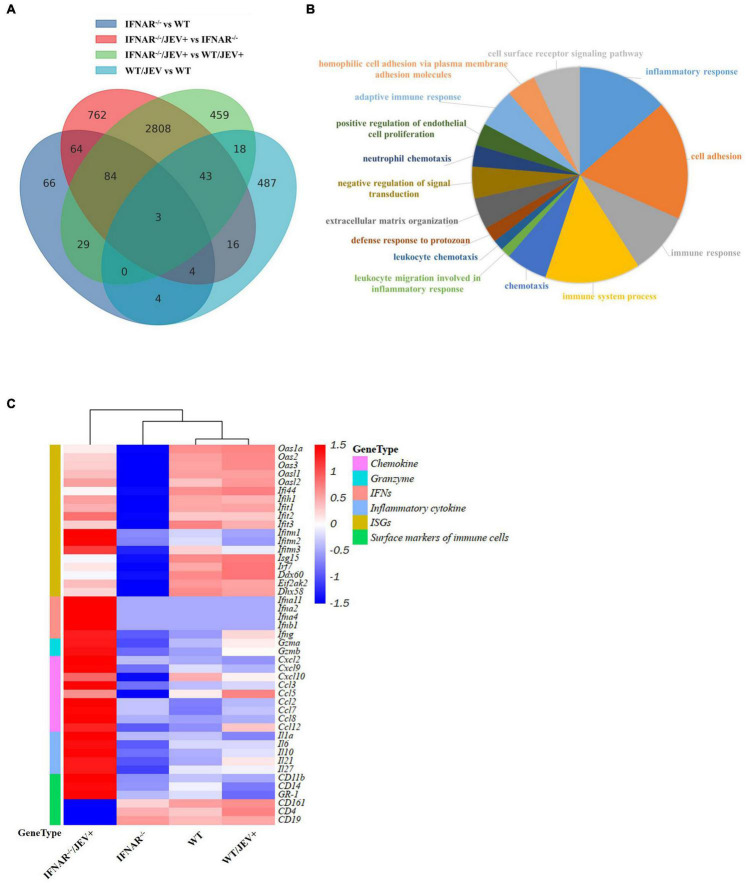
The transcript profile analysis of spleen in WT and IFNAR^–/–^ mice infected with SA14-14-2. **(A)** Venn diagram depicting the differentially expressed genes unique or common at comparable groups mock-IFNAR^–/–^ mice vs. mock-WT mice, infected-IFNAR^–/–^ mice vs. mock-IFNAR^–/–^ mice, infected-IFNAR^–/–^ mice vs. infected-WT mice, and infected-WT mice vs. mock-WT mice. **(B)** Enriched Gene Ontology terms in the biological process category among common genes only was comparable in groups infected-IFNAR^–/–^ mice vs. mock-IFNAR^–/–^ mice and infected-IFNAR^–/–^ mice vs. infected-WT mice. The corresponding color section represented the different biological processes. **(C)** Heat map analysis of differentially expressed key genes related to immune response. Each row showed the relative expression level for a single gene, and each column showed the average expression level of three biological repetition samples.

To further understand the biological process involved in SA14-14-2 infection, a systematic analysis of the transcription profile in spleens of mock- and SA14-14-2-infected-IFNAR^–/–^ and WT mice was performed by using RNA-sequencing (RNA-seq) at 4 dpi. Genes showing ≥ |2|-fold change in expression with *p* ≤ 0.05 were identified as the differentially expressed genes (DEGs). Venn diagram showed 2,808 common DEGs only for the infected-IFNAR^–/–^ mice vs. the mock-IFNAR^–/–^ mice and infected-WT mice (Figure 3A). Then, we selected these common DEGs for enrichment analysis of gene function. GO analysis revealed that these DEGs’ biological processes were mainly involved in inflammatory, immune system, immune, and cell adhesion (Figure 3B).

The interested DEGs involved in immune response processes, including chemokine, granzyme, IFNs, inflammatory cytokines, ISGs, and surface markers of immune cells, were assessed to confirm the enrichment results further. Heat maps showed that the genes in mock- and SA14-14-2-infected-WT mice were one cluster, along with genes in mock-IFNAR^–/–^ mice in one large cluster, and genes in JEV SA14-14-2-infected-IFNAR^–/–^mice were the other one large cluster (Figure 3C), indicating that the strong immune response was induced by JEV SA14-14-2 infecting IFNAR^–/–^mice, and no significant immune response was induced by JEV SA14-14-2 infecting WT mice. The subset with up-regulated genes in SA14-14-2-infected-IFNAR^–/–^mice contained type I and II IFNs genes (Ifna2, Ifna4, Ifna11, Ifnb1, and Ifng), the inflammatory cytokines genes (il-1a, il-10, il-21, il-27, and il-6), chemokines genes (Ccl2, Ccl3, Ccl7, Ccl8, Ccl9, Cxcl2, and Cxcl9), ISGs (Ifitm1 and Ifitm2) and inflammatory cell marker genes (GR-1, CD11b, and CD14) (Figure 3C and Supplementary Table 2). The subset with down-regulated genes in SA14-14-2-infected-IFNAR^–/–^mice contained leukocyte marker genes (CD19, CD4, and CD161) (Figure 3C and Supplementary Table 2). The subsets with down-regulated genes in mock-infected IFNAR^–/–^mice mainly contained ISGs, except for Ifitm1 and Ifitm2, in which the expression levels of ISGs were down-regulated or no significant change in SA14-14-2-infected-IFNAR^–/–^mice compared with that in mock-WT mice (Figure 3C and Supplementary Table 2). Collectively, these results indicated that the IFNAR^–/–^mice maintained low-level expression of ISGs, produced a severe inflammatory response, and reduced adaptive immune cells (CD4^+^ T and B cell) in response to SA14-14-2 infection.

### The Role of IFN-I System in Response to IFNAR^–/–^ Mice Infection With SA14-14-2

**FIGURE 4:**
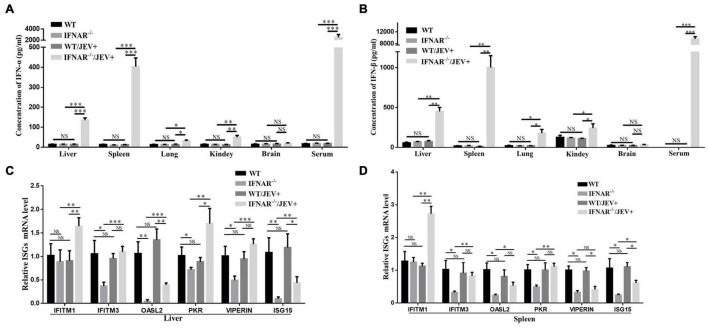
Effects of IFNAR deficiency on the IFN-I pathway after JEV vaccine strain SA14-14-2 infection. **(A,B)** Corresponding protein levels of IFN-α and IFN-β in each organ with significant viral replication and serum were measured by ELISA. The mRNA expression levels of ISGs in the liver **(C)** and spleen **(D)** were measured by using real-time PCR. All the data represent mean ± SD of biological triplicates from at least three independent experiments. The Student’s *t*-test performed statistical analyses. *P < 0.05, ^**^P < 0.01, ^***^P < 0.001. NS, no significance. N/D, not detectable.

To investigate the molecular mechanism of enhanced virulence and peripheral pathogenicity of SA14-14-2 virus infection, the effect of IFNAR deficiency on type I interferon (IFN) induction was determined by ELISA in the infected mice at 4 dpi. As shown in Figures 4A,B, the levels of IFN-α and IFN-β in serum and all tested organs, excluding brain, were significantly higher in the SA14-14-2-infected IFNAR^–/–^ mice, compared with those in SA14-14-2-infected WT mice or uninfected IFNAR^–/–^ mice, and no significant differences in these levels were observed among SA14-14-2-infected WT mice, uninfected WT mice, and uninfected IFNAR^–/–^ mice. The results indicated that IFNAR deficiency profoundly promoted IFN-α/β induction in response to SA14-14-2 strain infection. Besides, the expression of IFN-α and IFN-β in the tested organs was consistent with the peripheral tissue distribution of the SA14-14-2 strain and suggested that SA14-14-2 strain replication induced high levels of IFN-Is in IFNAR^–/–^ mice.

Furtherly, the effect of IFNAR deficiency on IFN-stimulated genes (ISG) induction was observed during SA14-14-2 strain infection. According to a previous study involving flavivirus infection (Schoggins, 2014), the mRNA levels of six key ISGs, i.e., IFITM1, IFITM3, OASL2, PKR, VIPERIN, and ISG15, were analyzed by real-time PCR in the spleen and liver. These two organs were selected because the spleen and liver suffered significant tissue damage, accompanied by high expression levels of IFN-α and IFN-β in this study. Compared with uninfected IFNAR^–/–^ mice, SA14-14-2-infected IFNAR^–/–^ mice showed a significant upregulation in the mRNA expression levels of all tested ISGs in the liver, and the mRNA expression levels of all tested ISGs, except for VIPERIN, were significantly upregulated in the spleen (Figures 4C,D). However, compared with SA14-14-2-infected WT mice, SA14-14-2-infected IFNAR^–/–^ mice showed significant ISG upregulation (IFITM1 and PKR) and downregulation (OASL2 and ISG15) in the liver (Figure 4C) as well as upregulation (IFITM1) and downregulation (VIPERIN and ISG15) in the spleen (Figure 4D). Compared with uninfected WT mice, SA14-14-2-infected WT mice showed no significant differences in the mRNA expression levels of the six tested ISGs, whereas the uninfected IFNAR^–/–^ mice exhibited a significant downregulation in the mRNA expression levels of most key ISGs, all except for IFITM1 (Figures 4C,D). These results suggested that the SA14-14-2 strain could still induce the upregulation of ISGs in IFNAR^–/–^ mice; however, IFNAR deficiency also contributed to the relatively low induction levels of ISGs in response to SA14-14-2 strain infection.

### SA14-14-2 Infection Significantly Increases the Expression of Peripheral Inflammatory Cytokines in IFNAR^–/–^ Mice

**FIGURE 5:**
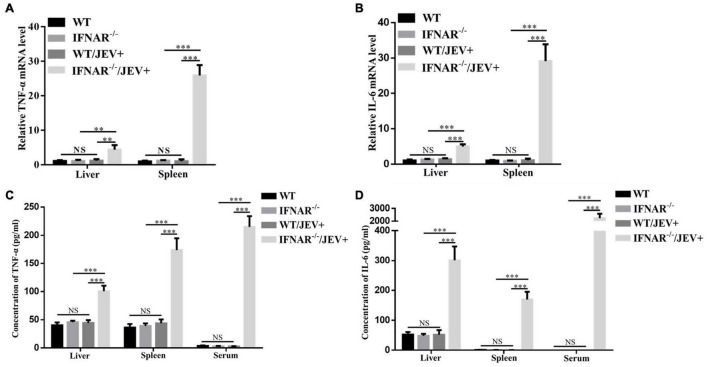
The expression level of inflammatory cytokines TNF-α and IL-6 significantly increased in IFNAR^–/–^ mice infected with SA14-14-2. **(A,B)** WT and IFNAR^–/–^ mice were intraperitoneally infected with JEV SA14-14-2 at 4 dpi, followed by real-time PCR for the mRNA levels of TNF-α and IL-6 in liver and spleen. **(C,D)** Corresponding protein levels TNF-α and IL-6 in liver and spleen and serum were measured by ELISA. All the data represent mean ± SD of biological triplicates from at least three independent experiments. The Student’s *t*-test performed statistical analyses. ^**^P < 0.01, ^***^P < 0.001, NS, no significance.

To confirm whether the increased pathogenicity of SA14-14-2 strain to IFNAR^–/–^ mice is associated with inflammatory responses, the levels of key inflammatory cytokines were measured in the liver and spleen. First, the transcript levels and protein expression of TNF-α and IL-6 were examined using real-time PCR and ELISA. The mRNA expression levels (Figures 5A,B) and protein expression (Figures 5C,D) of TNF-α and IL-6 in the livers and spleens were significantly upregulated in IFNAR^–/–^ mice compared with the WT counterparts infected with SA14-14-2. Interestingly, IFNAR deficiency dramatically increased the protein expression levels of TNF-α and IL-6 in the serum of mice infected with SA14-14-2 compared with those in WT mice (Figures 5C,D). Compared with those in uninfected WT mice, neither the transcript levels nor protein expression levels of TNF-α and IL-6 in organs and serum showed no significant difference in SA14-14-2-infected WT mice and uninfected WT mice (Figure 5). These results indicated that TNF-α and IL-6 production was robustly induced by SA14-14-2 strain infection in the absence of IFNAR.

**FIGURE 6:**
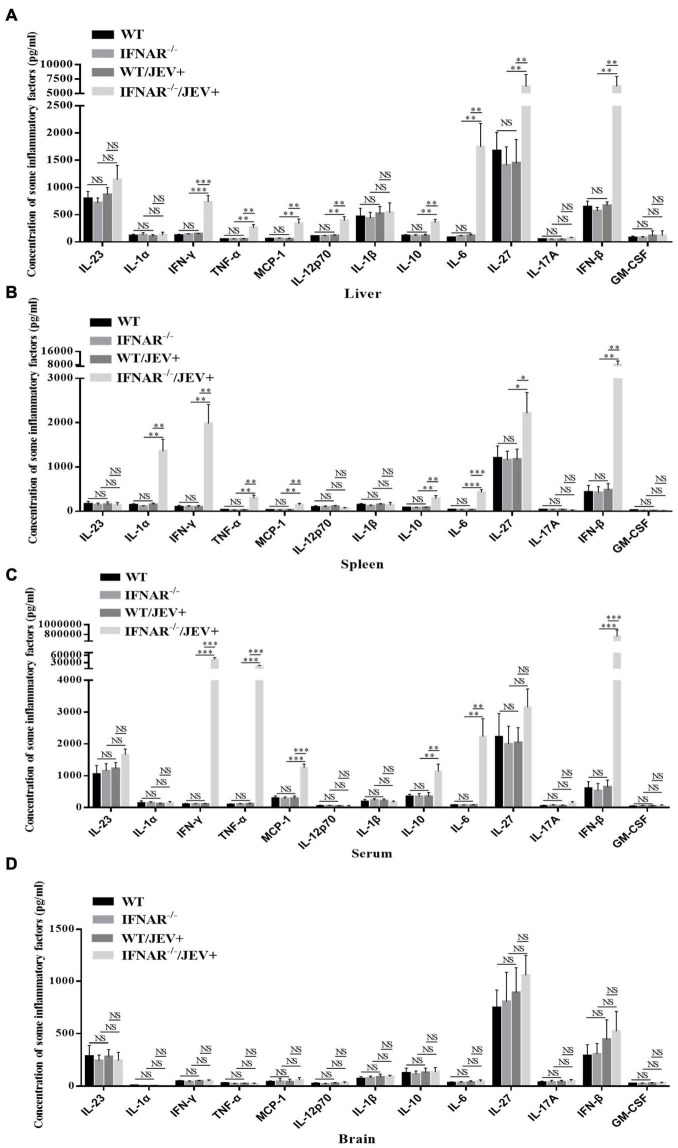
SA14-14-2 strain infection induced significant up-regulation of inflammatory cytokines in IFNAR^–/–^ mice. The concentration of inflammatory cytokines in the liver **(A)**, spleen **(B)**, serum **(C)**, and brain **(D)** were measured at 4 dpi by using a LEGENDplex™ Mouse Inflammation Panel (13-plex) with a filter plate (BioLegend). All the data represent mean ± SD of biological triplicates from at least three independent experiments. The Student’s *t*-test performed statistical analyses. *P < 0.05, ^**^P < 0.01, ^***^P < 0.001, NS, no significance.

For a more comprehensive and in-depth understanding of the inflammatory response caused by infection with the SA14-14-2 strain, a multiplex assay simultaneous quantification of 13 mouse inflammatory cytokines was applied. The results indicated that the expression levels of IFN-γ, INF-α, MCP-1, IL-10, IL-6, and IFN-β were significantly higher in all tested samples, especially the serum, from SA14-14-2-infected IFNAR^–/–^ mice (Figures 6A–C). However, in the brain, the expression levels of all tested inflammatory cytokines were not significantly different in SA14-14-2-infected IFNAR^–/–^ mice compared with the other experimental mice (Figure 6D). The results showed that IFNAR deficiency mainly caused severe systemic inflammation instead of neuroinflammatory damage to SA14-14-2 strain infection.

### Immune Cells Infiltrating Liver and Spleen in Response to SA14-14-2 Infection

**FIGURE 7:**
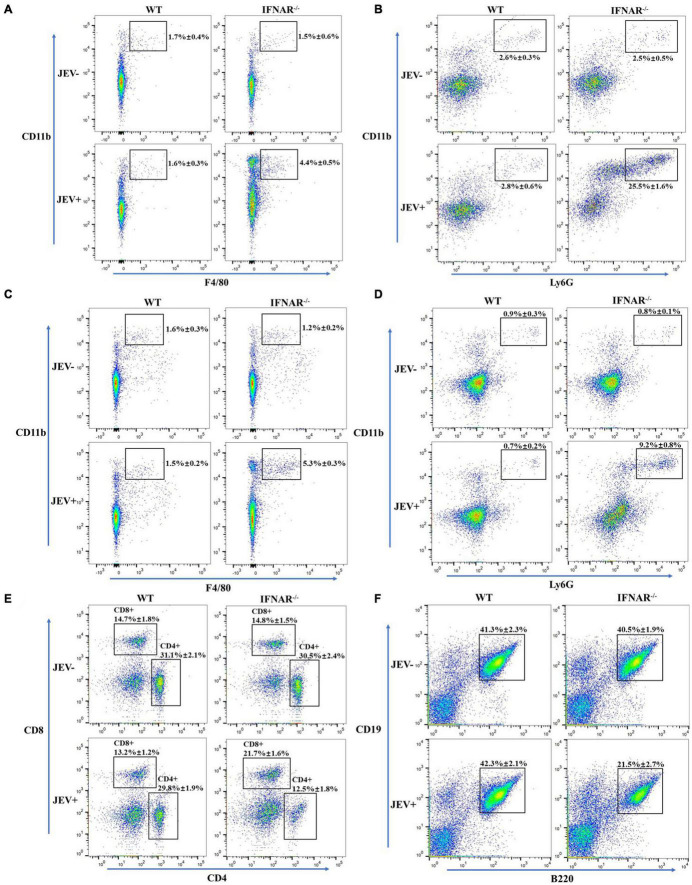
Inflammatory cells infiltrate the liver and spleen in response to the SA14-14-2 strain. Flow cytometry was performed on cells isolated from the liver and spleen of SA14-14-2 strain infected IFNAR^–/–^ mice at 4 dpi. **(A,B)** The populations of F4/80^+^CD11b macrophages **(A)** and Ly6G^+^CD11b neutrophils **(B)** were significantly increased in the liver of infected IFNAR^–/–^ mice. **(C,D)** The populations of F4/80^+^CD11b macrophages **(C)** and Ly6G^+^CD11b neutrophils **(D)** were significantly increased in the spleen of infected IFNAR^–/–^ mice. **(E,F)** The populations of CD4^+^ T and B cells were significantly decreased, and the population of CD8^+^ T was significantly increased in the spleen of infected IFNAR^–/–^ mice. All the data represent mean ± *SD* of biological triplicates from at least three independent experiments.

The above data suggested that the livers and spleens of IFNAR^–/–^ mice infected with the SA14-14-2 strain exhibited histopathological lesions and severe peripheral inflammatory responses. Cells were isolated from the liver and spleen, and the cell populations were analyzed using flow cytometry. SA14-14-2-infected IFNAR^–/–^ mice showed significantly higher cellular infiltration levels than SA14-14-2-infected WT mice, with larger percentages of CD11b^+^Ly6G^+^ neutrophils and CD11b^+^F4/80^+^ macrophages, gated from the CD45^hi^ infiltrating population in both liver (Figures 7A,B) and spleen (Figures 7C,D). Moreover, the percentages of acquired immune cells in spleens were measured. The results showed that CD8^+^ T cells gated from the CD45^hi^ infiltrating population in the spleen. However, the percentages of CD4^+^ T and B cells in SA14-14-2-infected IFNAR^–/–^ mice were significantly lower than those in SA14-14-2-infected WT mice (Figures 7E,F). The recruitment and activation of neutrophils and monocyte/macrophages engaged in cytokine storm indicated vigorous inflammation response in SA14-14-2-infected IFNAR^–/–^ mice. Moreover, a declining proportion of CD4^+^ T cells and B cells suggested lymphoid depletion and immune injury caused by the SA14-14-2 virus in IFNAR^–/–^ mice.

## Discussion

Japanese encephalitis (JE) is one of the most epidemic viral encephalitides in humans and animals and threatens global public health. Although the clinical features of JEV-infected individuals vary from mild symptoms to severe meningitis, the risk of virus encephalitis may be low in the clinic with 1% probability (Halstead and Jacobson, 2013). The peripheral immune response plays an essential role in defending the development of minor illness during JEV infection without encephalitis and fighting the transmission of JEV from the periphery to the CNS (Sharma et al., 2021). Here, we investigated the peripheral pathological response to JEV infection in BSL-2 containment by a model of IFNAR^–/–^/JEV SA14-14-2. Our results revealed that the SA14-14-2 strain resulted in lethal peripheral inflammatory responses in infected IFNAR^–/–^ mice, and the IFNAR^–/–^/JEV SA14-14-2 model may be used to assess the peripheral pathogenesis of JEV infection.

In this study, the upregulation of IFN-α/β and ISGs in SA14-14-2-infected IFNAR^–/–^ mice further confirmed that IRF3/IRF7 not only directly induce the production of IFN-α/β but also can directly induce the expression of ISGs independently of IFNAR signaling (Honda et al., 2005; Honda and Taniguchi, 2006; Schmid et al., 2010). Meanwhile, the results showed that most ISGs were downregulated in uninfected IFNAR^–/–^ mice compared with uninfected WT mice, and the relatively low levels of ISGs were induced in infected IFNAR^–/–^ mice. These suggested that the expression of ISGs was inhibited in IFNAR^–/–^ mice, and low levels of ISGs would cause widespread virus replication. A relatively low virus load was observed in the tested organs, and no virus was detected in the brain induced by the SA14-14-2 strain, which suggested that other antiviral mechanisms independent of the function of IFN-I may exist. The most likely mechanism for the inhibition of virus replication might be that IFN-γ response plays a compensatory antiviral role to IFN-α/β.

Previous studies had confirmed that the mice both lacking the capacity to respond to IFN-α/β and IFN-γ were highly susceptible to JEV SA14-14-2, YFV, and DENV infection (Meier et al., 2009; Calvert et al., 2014; Sarathy et al., 2015), and G129 mice (IFN-γ receptor-deficient) are largely resistant to JEV candidate vaccine, YFV and DENV infection (Liang et al., 2009; Meier et al., 2009; Sarathy et al., 2015). IFN-γ was critical in suppression of JEV growth in the CNS but not required for effective clearance of virus from extraneural tissues (Larena et al., 2013), which may throw light on no appearance of classic viral encephalitis (without neurologic disorders and damage) in infected IFNAR^–/–^ mice due to the up-regulated IFN-γ levels detected; however, further research is needed to explore the exact mechanism. Compared with AG129 mice infected with SA14-14-2, where a lower dose of virus caused death with higher titers of infectious virus in the brain (Calvert et al., 2014), our experiment showed that only high titer of SA14-14-2 infection was lethal, and no virus was detected in the brain of IFNAR^–/–^ mice. These results suggested that IFNAR^–/–^ mice had only moderate sensitivity to SA14-14-2 infection.

Antiviral pro-inflammatory responses are essential for controlling viral replication and clearance. However, in severe cases, excessive inflammatory responses induced by virus infection would contribute to disease pathogenesis and even death (Brittle et al., 2004). Some studies have reported that increased inflammatory cytokines like IL-6, TNF-α, and chemokines like CCL2 (MCP-1), CCL5 induced irreversible damage and were associated with increased mortality of mice (Ghoshal et al., 2007; Chen et al., 2010). Our results showed that several key inflammatory cytokines, including MCP-1, IL-6, TNF-α, and IFN-γ, were sharply promoted in visceral tissues and serum of IFNAR^–/–^ mice infected with SA14-14-2. Moreover, flow cytometry detected a high percentage of infiltrating neutrophils and macrophages in the spleen and liver, confirming a severe peripheral inflammation response to SA14-14-2 strain infection. Additionally, lymphoid depletion and acquired immune injury, accompanied by the reduced percentages of CD4^+^ T and B cells, were observed in the spleen of SA14-14-2-infected IFNAR^–/–^ mice, which was consistent with IFNAR^–/–^animals experimentally infected with Bluetongue virus or Zika virus (Dowall et al., 2016; Marin-Lopez et al., 2016). These results suggested that the replication of JEV SA14-14-2 in peripheral organs dramatically increased the expression of pro-inflammatory cytokines and infiltration of inflammatory cellular, contributing to severe damage, even to death.

In summary, immunopathogenesis and peripheral inflammation response during JEV SA14-14-2 strain infection in the IFNAR^–/–^mice model were evaluated. Our study may provide multiple disease parameters to evaluate clinical pathogenicity study including severe viscerotropic inflammation and pathological injury, especially spleen and liver. These results confirmed that the IFN-I plays an important function in maintaining vaccine safety of the SA14-14-2 strain. IFNAR^–/–^mice may provide a valuable model for studying immunopathogenesis and antiviral therapies.

## Data Availability Statement

The datasets presented in this study can be found in online repositories. The names of the repository/repositories and accession number(s) can be found below: NCBI SRA BioProject, accession no: PRJNA787393.

## Ethics Statement

The animal study was reviewed and approved by the Animal Ethics Committee of Lanzhou Veterinary Research Institute, Chinese Academy of Agricultural Sciences (Permit No. LVRIAEC-2019-012). All experimental mouse procedures were conducted following the Good Animal Practice Requirements of the Animal Ethics Procedures and Guidelines of the People’s Republic of China.

## Author Contributions

JL, ZJ, and JW conceived, designed the study, and critically revised the manuscript. JL, WJ, YF, XH, HJ, and GC performed the experiments, analyzed the data, and drafted the manuscript. JL wrote the manuscript. All authors read and approved the final manuscript.

## Conflict of Interest

The authors declare that the research was conducted in the absence of any commercial or financial relationships that could be construed as a potential conflict of interest.

## Publisher’s Note

All claims expressed in this article are solely those of the authors and do not necessarily represent those of their affiliated organizations, or those of the publisher, the editors and the reviewers. Any product that may be evaluated in this article, or claim that may be made by its manufacturer, is not guaranteed or endorsed by the publisher.

## Supplementary Material

Supplementary Figure 1 Comparative photomicrograph of the lung, kidney, and brain tissue sections from SA14-14-2 strain infected- and uninfected-WT and IFNAR^–/–^ mice. H&E; 20 × and 200 ×. Representative pictures of tissues were collected at 4 dpi and stained with hematoxylin and eosin. No significant pathological changes were observed in lung **(A)**, kidney **(B)**, and brain **(C)** collected from corresponding infected and uninfected mice.
