# Supplementary figures and images for: The Infection of the Japanese Encephalitis Virus SA14-14-2 Strain Induces Lethal Peripheral Inflammatory Responses in IFNAR Deficiency Mice
Source: Front Microbiol. 2022 Mar 3;12:823825. doi: 10.3389/fmicb.2021.823825 (PMC8928384; doi:10.3389/fmicb.2021.823825)

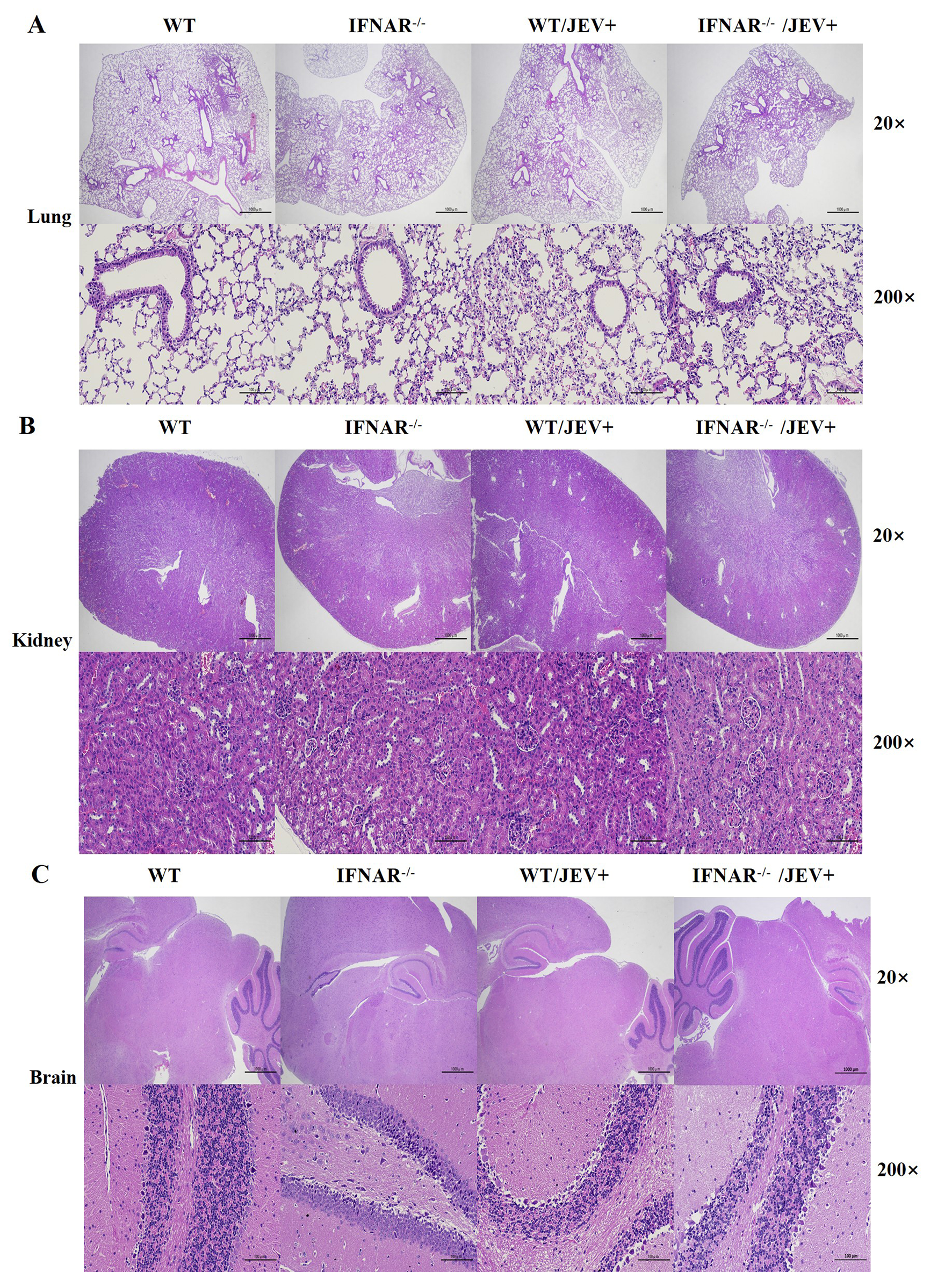

Supplement: Supplementary Figure 1 — Comparative photomicrograph of the lung, kidney, and brain tissue sections from SA14-14-2 strain infected- and uninfected-WT and IFNAR–/– mice. H&E; 20 × and 200 ×. Representative pictures of tissues were collected at 4 dpi and stained with hematoxylin and eosin. No significant pathological changes were observed in lung (A), kidney (B), and brain (C) collected from corresponding infected and uninfected mice. [file Image_1.TIF]
