# Supplementary material for: The Infection of the Japanese Encephalitis Virus SA14-14-2 Strain Induces Lethal Peripheral Inflammatory Responses in IFNAR Deficiency Mice
Source: Front Microbiol. 2022 Mar 3;12:823825. doi: 10.3389/fmicb.2021.823825 (PMC8928384; doi:10.3389/fmicb.2021.823825)
Supplement: Supplementary file 2 [file Table_1.docx]

**Table. S1** Primers used in this study

| Gene | Primer | Sequence (5’→3’) | References |
| --- | --- | --- | --- |
| *IL-6* | IL-6-F | ACCACGGCCTTCCCTACTTCAC | [1] |
|  | IL-6-R | TCCTCATTTCCACGATTTCCCAG |  |
| *TNF-α* | TNF-α-F | AGTCCGGGCAGGTCTACTTT | [2] |
|  | TNF-α-R | GTCACTGTCCCAGCATCTTGT |  |
| *ifitm1* | ifitm1-F | GACAGCCACCACAATCAACAT | PrimerBank |
|  | ifitm1-R | CCCAGGCAGCAGAAGTTCAT |  |
| *ifitm3* | ifitm3-F | CCCCCAAACTACGAAAGAATCA | PrimerBank |
|  | ifitm3-R | ACCATCTTCCGATCCCTAGAC |  |
| *Oasl2* | Oasl2-F | CCGTTCCCCGACCTGTATG | PrimerBank |
|  | Oasl2-R | CCTTCACCACCTTAATCACCCT |  |
| *pkr* | pkr-F | TGGATTGTCACACGAGTGCAT | PrimerBank |
|  | pkr-R | GTTGGGCTCACACTGTTCATAAT |  |
| *viperin* | viperin-F | TGCTGGCTGAGAATAGCATTAGG | PrimerBank |
|  | viperin-R | GCTGAGTGCTGTTCCCATCT |  |
| *isg15* | isg15-F | GGTGTCCGTGACTAACTCCAT | PrimerBank |
|  | isg15-R | TGGAAAGGGTAAGACCGTCCT |  |
| *β-actin* | β-actin-F | CCGTGAAAAGATGACCCAGATC | [3] |
|  | β-actin-R | CACAGCCTGGATGGCTACGT |  |
| JEV-E | JE3F1-F | CCCTCAGAACCG TCT CGG AA | [4] |
|  | JE3R1-R | CTATTCCCAGGTGTCAATATGCTGT |  |

1. Marin-Lopez, A.; Bermudez, R.; Calvo-Pinilla, E.; Moreno, S.; Brun, A.; Ortego, J. Pathological Characterization Of IFNAR(-/-) Mice Infected With Bluetongue Virus Serotype 4. *Int J Biol Sci* **2016**, *12*, 1448-1460, doi:10.7150/ijbs.14967.

2. Huang, P.; Li, S.; Shao, M.; Qi, Q.; Zhao, F.; You, J.; Mao, T.; Li, W.; Yan, Z.; Liu, Y. Calorie restriction and endurance exercise share potent anti-inflammatory function in adipose tissues in ameliorating diet-induced obesity and insulin resistance in mice. *Nutr Metab (Lond)* **2010**, *7*, 59, doi:10.1186/1743-7075-7-59.

3. Lim, S.M.; Jeong, J.J.; Kang, G.D.; Kim, K.A.; Choi, H.S.; Kim, D.H. Timosaponin AIII and its metabolite sarsasapogenin ameliorate colitis in mice by inhibiting NF-kappaB and MAPK activation and restoring Th17/Treg cell balance. *Int Immunopharmacol* **2015**, *25*, 493-503, doi:10.1016/j.intimp.2015.02.016.

4. Jeong, H.S.; Shin, J.H.; Park, Y.N.; Choi, J.Y.; Kim, Y.L.; Kim, B.G.; Ryu, S.R.; Baek, S.Y.; Lee, S.H.; Park, S.N. Development of real-time RT-PCR for evaluation of JEV clearance during purification of HPV type 16 L1 virus-like particles. *Biologicals* **2003**, *31*, 223-229, doi:10.1016/s1045-1056(03)00064-2.

**References**
