# Supplementary material for: The Infection of the Japanese Encephalitis Virus SA14-14-2 Strain Induces Lethal Peripheral Inflammatory Responses in IFNAR Deficiency Mice
Source: Front Microbiol. 2022 Mar 3;12:823825. doi: 10.3389/fmicb.2021.823825 (PMC8928384; doi:10.3389/fmicb.2021.823825)
Supplement: Supplementary file 3 [file Table_2.docx]

Table S2 Differentially-regulated genes related to immune response in spleen of mice with JEV SA14-14-2 infection^a^

| Genes | | | Fold change* (log_2_Foldchang) | | | |
| --- | --- | --- | --- | --- | --- | --- |
| Genbank Accession | Gene symbol | Gene description | IFNAR^i^/IFNAR^u^ | IFNAR^i^/WT^i^ | WT^i^/WT^u^ | IFNAR^u^/WT^u^ |
| **The interferon-stimulated genes (ISGs)** | |  |  |  |  |  |
| NM_145211.2 | Oas1a | 2'-5' oligoadenylate synthetase 1A | 6.79 | -2.35 | - | -8.83 |
| XM_006530325.3 | Oas2 | 2'-5' oligoadenylate synthetase 2 | 6.35 | -1.55 | - | -7.38 |
| NM_145226.2 | Oas3 | 2'-5' oligoadenylate synthetase 3 | 3.89 | - | - | -4.43 |
| XM_006530294.3 | Oasl1 | 2'-5' oligoadenylate synthetase-like 1 | 4.84 | - | - | -5.29 |
| NM_011854.2 | Oasl2 | 2'-5' oligoadenylate synthetase-like 2 | 4.25 | - | - | -3.85 |
| NM_133871.2 | Ifi44 | interferon-induced protein 44 | 5.87 | -2.23 | - | -7.83 |
| NM_008331.3 | Ifit1 | interferon-induced protein with tetratricopeptide repeats 1 | 5.33 | - | - | -5.38 |
| XM_006526705.2 | Ifit2 | interferon-induced protein with tetratricopeptide repeats 2 | 1.72 | - | - | -1.32 |
| XM_011247153.1 | Ifit3 | interferon-induced protein with tetratricopeptide repeats 3 | 3.12 | - | - | -3.85 |
| NM_026820.3 | Ifitm1 | interferon induced transmembrane protein 1 | 1.92 | 1.78 | - | - |
| NM_030694.1 | Ifitm2 | interferon induced transmembrane protein 2 | 1.20 | 1.13 | - | - |
| NM_025378.2 | Ifitm3 | interferon induced transmembrane protein 3 | 1.85 | - | - | -1.16 |
| NM_015783.3 | Isg15 | ISG15 ubiquitin-like modifier | 2.39 | - | - | -3.66 |
| NM_011163.4 | Eif2ak2 | eukaryotic translation initiation factor 2-alpha kinase 2 | 1.36 | - | - | -1.50 |
| XM_017312698.1 | Ddx60 | DEAD (Asp-Glu-Ala-Asp) box polypeptide 60 | 1.64 | -1.04 | - | -2.53 |
| XM_006536214.1 | irf7 | interferon regulatory factor 7 | 2.40 | -1.02 | - | -2.94 |
| NM_030150.2 | Dhx58 | DEXH (Asp-Glu-X-His) box polypeptide 58 | 1.84 | - | - | -2.32 |
| **The cytokines genes** | |  |  |  |  |  |
| NM_008333.2 | Ifna11 | interferon alpha 11 | inf** | inf | - | - |
| NM_010503.2 | Ifna2 | interferon alpha 2 | inf | inf | - | - |
| NM_010504.2 | Ifna4 | interferon alpha 4 | inf | inf | - | - |
| NM_010510.1 | Ifnb1 | interferon beta 1 | inf | inf | - | - |
| NM_008337.4 | Ifng | interferon gamma | 2.05 | 1.05 | - | - |
| NM_010370.2 | Gzma | granzyme A | 3.85 | 1.99 | - | - |
| NM_013542.2 | Gzmb | granzyme B | 7.92 | 4.82 | 1.93 | -1.15 |
| NM_021274.2 | Cxcl10 | chemokine (C-X-C motif) ligand 10 | 1.61 | - | - | -1.29 |
| NM_009140.2 | Cxcl2 | chemokine (C-X-C motif) ligand 2 | 3.37 | 3.95 | - | - |
| NM_008599.4 | Cxcl9 | chemokine (C-X-C motif) ligand 9 | 3.21 | 2.65 | - | - |
| NM_011331.2 | Ccl12 | chemokine (C-C motif) ligand 12 | 4.90 | 1.84 | - | - |
| NM_011333.3 | Ccl2 | chemokine (C-C motif) ligand 2 | 1.91 | 2.00 | - | - |
| NM_011337.2 | Ccl3 | chemokine (C-C motif) ligand 3 | 3.18 | 2.32 | - | - |
| NM_013653.3 | Ccl5 | chemokine (C-C motif) ligand 5 | 1.28 | - | - | - |
| NM_013654.3 | Ccl7 | chemokine (C-C motif) ligand 7 | 2.72 | 2.70 | - | - |
| NM_021443.3 | Ccl8 | chemokine (C-C motif) ligand 8 | 4.82 | 4.80 | - | - |
| NM_011338.2 | Ccl9 | chemokine (C-C motif) ligand 9 | 1.45 | 1.56 | - | - |
| NM_010548.2 | Il10 | interleukin 10 | 5.50 | 3.83 | - | - |
| XM_006498793.3 | Il1a | interleukin 1 alpha | 1.29 | 1.49 | - | - |
| NM_021782.3 | Il21 | interleukin 21 | 5.04 | 2.52 | 1.33 | - |
| XM_006507857.1 | Il27 | interleukin 27 | 4.12 | 2.31 | - | -1.71 |
| NM_031168.2 | Il6 | interleukin 6 | 4.44 | 2.26 | - | - |
| **Surface markers genes of immune cells** | |  |  |  |  |  |
| NM_001082960.1 | CD11b (ItgaM) | integrin alpha M | 1.82 | 1.67 | - | - |
| NM_009841.4 | CD14 | CD14 antigen | 2.55 | 2.74 | - | - |
| NM_009841.4 | CD14 | CD14 antigen | 2.55 | 2.74 | - | - |
| NM_001310438.1 | GR-1 (Ly6g) | lymphocyte antigen 6 complex, locus G | 2.59 | 3.25 | - | - |
| NM_008527.2 | CD161 (Klrb1c) | killer cell lectin-like receptor subfamily B member 1C | -2.69 | -3.32 | - |  |
| NM_013488.2 | CD4 | CD4 antigen | -1.11 | -1.27 | - | - |
| XM_006507282.3 | CD19 | CD19 antigen | -2.52 | -2.46 | - | - |

^a^The upregulated ISGs and cytokines genes and significantly different surface markers genes of immune cells were selected in SA14-14-2 infected IFNAR^-/-^ mice, compared with uninfected- IFNAR^-/-^ mice, at 4 dpi.

* Superscripts “i” and “u” represent infected and uninfected mice, respectively

** inf represent that the mRNA expression wasn’t detected in comparable group
